# Effect of Curing Agent and Temperature on the Rheological Behavior of Epoxy Resin Systems

**DOI:** 10.3390/molecules17078587

**Published:** 2012-07-17

**Authors:** Chenhui Zhao, Guangcheng Zhang, Lei Zhao

**Affiliations:** School of Natural and Applied Science, Northwestern Polytechnical University, Xi’an 710072, China; Email: zhangguc@nwpu.edu.cn (G.Z.); irayson@163.com (L.Z.)

**Keywords:** epoxy resins, cure kinetics, curing agent, rheological behavior

## Abstract

The effect of curing agent (6610) content and temperature on the rheological behavior of the epoxy resin CYD-128 was studied by DSC analysis and viscosity experiments. The results show that the resin system meets the requirements of processing technology. A complete reaction occurs when the curing agent content (40 parts per hundred resin, phr) is a little higher than the theoretical value (33.33 phr), while the degree of reaction of the resin system is reduced when the curing agent content is lower (25.00 phr) than theoretical value. However, excessive curing agent (50.00 phr) results in a lower reaction rate. Curing agent content has little influence on gel time when curing agent content exceeded 33.33 phr and the temperature was less than 70 °C. The isothermal viscosity-time curves shift towards the –x axis when the temperature rises from 50 °C to 80 °C. Meanwhile, higher temperature results in higher reaction rates.

## 1. Introduction

Epoxy resins are commonly used as polymeric matrices in high-performance composites [1]. Epoxy resins is used extensively due to their low shrinkage, high adhesive strength, adjustable functional structure and better mechanical performance, especially in fields that require high strength and excellent performance such as wind blades and aerospace engineering [2,3,4,5].

There are many steps involved in the production process, from the selection of raw materials to the manufacture of the finished products. Of the many steps involved, the processing step plays a pivotal role in determining the quality of the final products [6,7]. The mechanism and kinetics of cure determine the network morphology, which, in turn, dictates the physical and mechanical properties of the cured product. Thus, understanding the cure kinetics of thermosets is essential for process development and quality control [8]. Perhaps the most important properties of polymeric materials with respect to their processing behavior are the rheological behavior. Viscosity control during processing of thermosets is particularly critical because the viscosity varies not only with temperature and flow conditions, but also with time because of polymerization reactions. Therefore, a comprehensive understanding of the relationship between the curing kinetics and rheological behavior is necessary to effectively control the cure process and to optimize the processing schedules and the properties of the finished product.

Despite the extensive literature on the polymerisation/curing of epoxy resins with amines, detailed studies on the effects of curing agent ratio on the curing kinetic and viscosity of a special system are generally not available [9,10,11,12,13,14,15,16,17,18,19,20,21]. The present study was carried out in order to determine the relationship between curing agent ratio and rheological behavior of the resin system.

## 2. Results and Discussion

### 2.1. Effect of Curing Agent Content on Curing Characteristics

**Figure 1:**
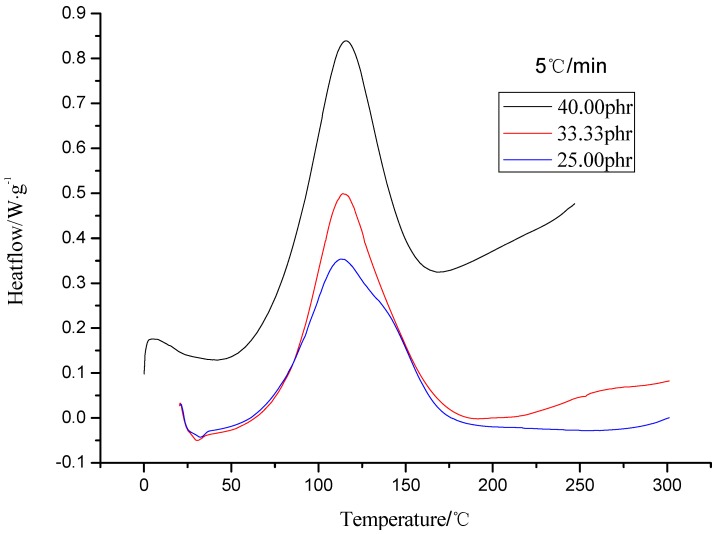
DSC curves at different curing agent content.

DSC curves of resin systems with 25, 33.3 and 40 phr of curing agent are shown in Figure 1, and the heats of reactions (*∆H*), start temperature (*Ts*), end temperature (*Te*) and peak temperature (*Tp*) are reported in Table 1.

**Table 1:** DSC results of different curing agent phr.

Phr of curing agent	*Ts *(°C)	*Tp *(°C)	*Te *(°C)	*∆H *(J/g)
40.00	37.97	113.54	174.12	335.7
33.33	43.07	116.97	209.79	315.4
25.00	47.23	122.57	215.10	275.6

It can be found there is wide exothermic peak, as shown in Figure 1, and we can also obtain the information from Table 1 that *∆H* ranges from 275.6 to 335.7 J/g which implies a low heat release. The combination of these two results indicate that the resin system meet the processing technology requirements. *∆H* from Table 1 indicates that a complete reaction occurs when the curing agent content (40 phr) is higher than theoretical value (33.33 phr), while the degree of reaction of the resin system reduces when the curing agent content is lower (25 phr) than the theoretical value.

### 2.2. Effect of Temperature and Curing Agent Content on Resin System Viscosity

**Figure 2:**
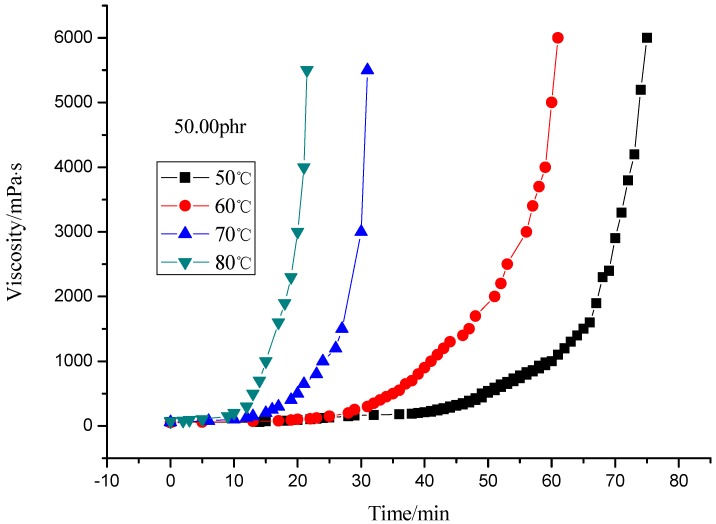
Viscosity of resin system with 50.00 phr curing agent at different temperature.

**Figure 3:**
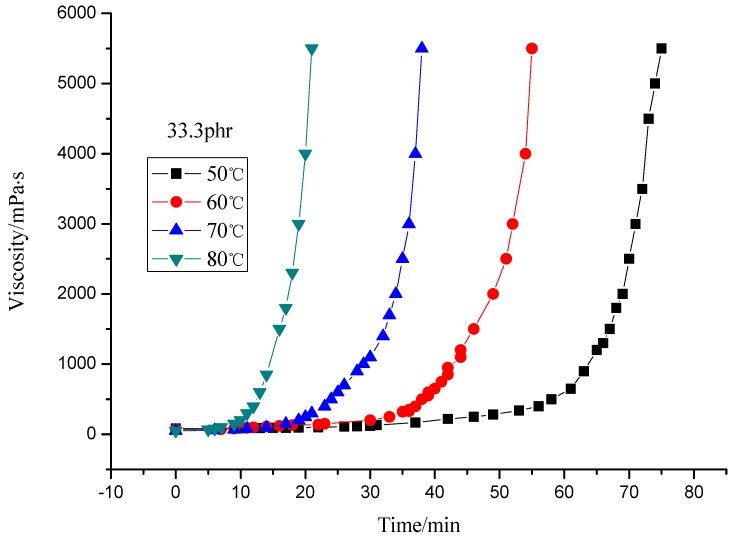
Viscosity of resin system with 33.33 phr curing agent at different temperature.

**Figure 4:**
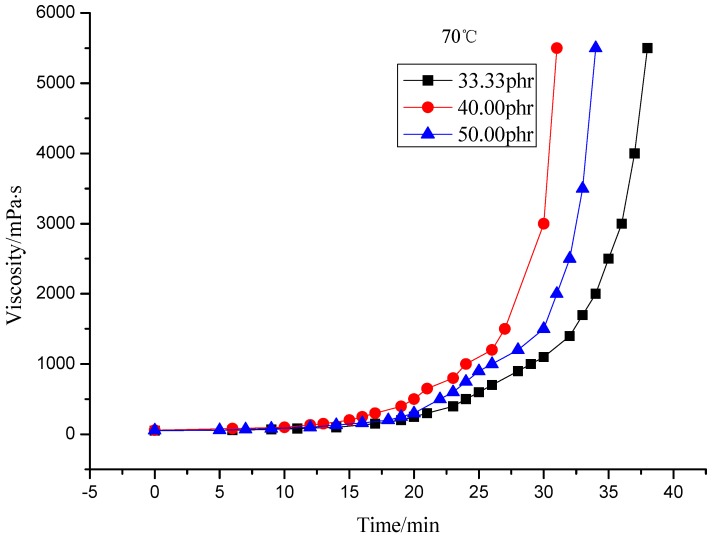
Viscosity of resin system with different curing agent content at 70 °C.

**Figure 5:**
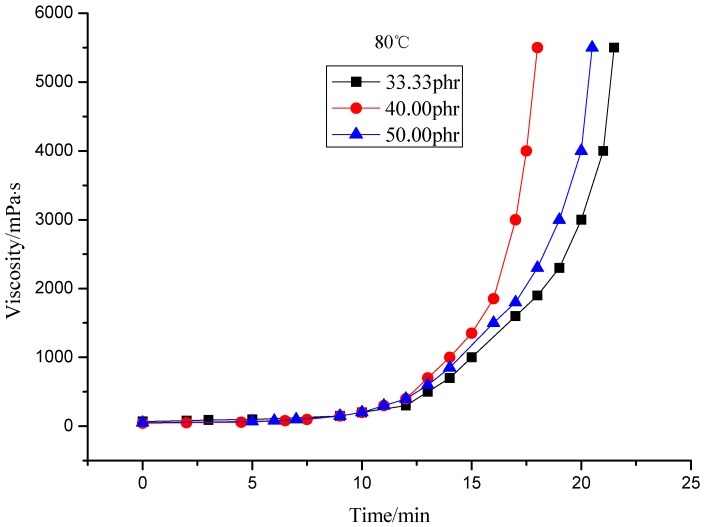
Viscosity of resin system with different curing agent content at 80 °C.

This paper studied the isothermal viscosity-time curves over a temperature range from 50 to 80 °C for different curing agent contents (33.33, 40.00 and 50.00 phr). The results are shown in Figure 2, Figure 3, Figure 4, Figure 5. The curves displayed in Figure 2 and Figure 3 show the same shape, which suggests the same cure kinetic mechanism for each system. It can also be observed that all the curves show the same functional form, only shifted by a constant factor along the x (time) axis. It follows that all the curves at different temperatures should be the same shape by simply shifting each curve along the x (time) axis relative to a curve at an arbitrary reference temperature by a shift factor [1]. Meanwhile, the slopes of the curves are different from each other. The slope increases as the temperature rises, which represents the reaction rate of the system. The difference of the slope indicates the temperature dependence of the reaction. Higher temperature results in higher reaction rate. This is similar to the report by Mounif *et al. *[22].

Both Figure 4 and Figure 5 indicate that the viscosity of the system with 40 phr curing agent rises faster than that with 33.33 phr (theoretical value), which may be caused by excess curing agent, but the viscosity rise rate slows when more curing agent is added (50 phr), the reason may be that excessive curing agent dilutes the resin which in turn hinders the crosslinking reaction.

### 2.3. Effect of Curing Agent Content on Gel Time

**Figure 6:**
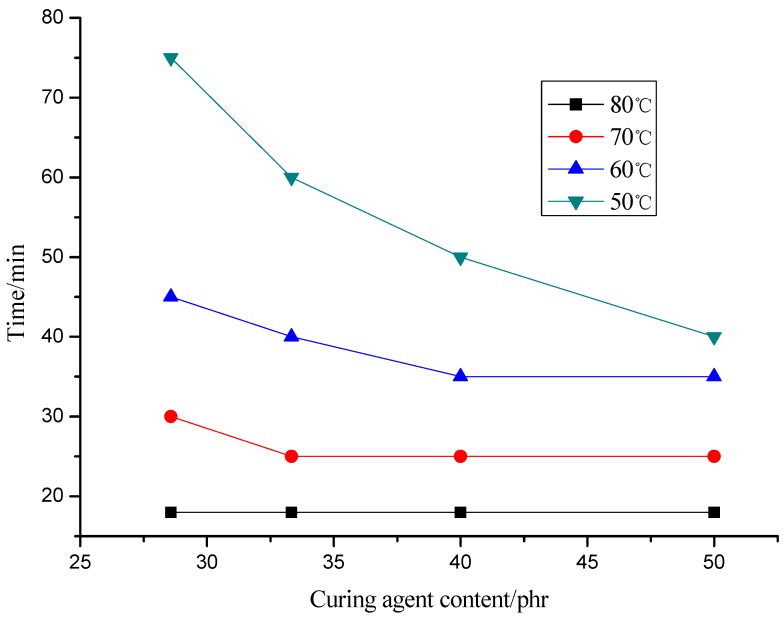
Relationship between gel time and curing agent content.

Gel time is one of the most important parameters in any molding process. The gel time reflects the activity of system according to temperature. Measurements are performed in constant temperature. In this process, the influence of curing agent content (28.57, 33.33, 40.00 and 50.00 phr) on the gel time of the epoxy resin at different temperatures (50, 60, 70 and 80 °C) was tested (Figure 6). 

It shows that the curing agent content has little effect on gel time when the temperature is less than 70 °C and curing agent content exceeds 33.33 phr. On the contrary, when the temperatures are below 70 °C and curing agent content is less than 33.33 phr, gel time increases with the decreasing curing agent content at the same temperature; gel time shortens with the rising of constant temperature at the same phr of curing agent.

## 3. Experimental

### 3.1. Experiment Preparation

The rheological behavior of the system diglycidyl bisphenol-A (CYD-128, Viscosity: 11,000–14,000 mpa·s at 25 °C, Exoxy value: 0.51–0.54 mol/100 g) and amine curing agent (6610, amine value: 280 ± 20 mg KOH/g) was studied by both differential scanning calorimetry (DSC) and rheometry. The components were mixed at room temperature by an electric mixer (Type: 6511, Shanghai Specimen Model Factory, Shanghai, China) at room temperature and degassed under vacuum for 15 min. The resulting resin mixtures were either tested immediately or stored at −40 °C in a dry-box containing silica gel. When samples were removed from the refrigerator they were allowed to warm to room temperature before being placed in hermetically sealed aluminum pans.

### 3.2. Experimental Procedures

Kinetic studies of samples prepared using 25.00, 33.33 and 50.00 phr of curing resin which were sealed in aluminum pans with weights in the range 5–15 × 10^−3 g were carried out using ^DSC (TA Instrument-2910, New Castle, DE, USA) at the heating rate of 5 °C/min, and all kinetic studies were performed under a nitrogen atmosphere The viscosities of the resin system containing either 33.33, 40.00 or 50.00 phr of curing agent and subjected to isothermal curing at different temperatures (50, 60, 70 and 80 °C) were measured by a rotational viscometer (NDJ-7, Shanghai Changji Geological Instruments Co., Ltd., Shanghai, China). The gel time is determined by wire drawing. 

## 4. Conclusions

In this work, the effect of the curing agent content on cure kinetics and rheological behavior of a resin system was studied. The results indicate that the resin system meet the requirements of processing technology; a complete reaction occurs when the curing agent content is higher (40 phr) than theoretical value and excessive curing agent (50.00 phr) results in lower reaction rates. The isothermal viscosity-time curves shift towards the –x axis when the temperature rises. Curing agent content has little influence on gel time when the temperature is lower than 70 °C and curing agent content exceeds 33.33 phr.
